# Tapeworms of freshwater fishes in North America: an integrative review of taxonomy, phylogeny, host specificity, and research priorities

**DOI:** 10.3389/fvets.2025.1661118

**Published:** 2025-10-02

**Authors:** Tomáš Scholz, Roman Kuchta

**Affiliations:** Institute of Parasitology, Biology Centre, Czech Academy of Sciences, České Budějovice, Czechia

**Keywords:** diversity, evolution, host-parasite associations, distribution, Cestoda, ray-finned fish, Nearctic region

## Abstract

Tapeworms (Cestoda) are a diverse group of parasitic flatworms that are highly specialized in a parasitic lifestyle. The freshwater fish tapeworms of North America have been relatively well studied since 1855, although their diversity is limited compared to other regions. Most knowledge was gained in the 20th century, with research declining in recent decades, although some groups have recently been revised based on morphological and molecular data. This review updates the current understanding of species diversity, phylogenetic relationships and host–parasite interactions based on a critical re-evaluation of the earlier records. The new data can also inform fisheries management, invasive species control and public health surveillance. Currently, 130 valid adult species are known in six orders, most of which are native to the Nearctic, with the USA having the greatest diversity (121 species). The vast majority of North American fish tapeworms exhibit narrow host specificity, with almost three quarters specializing in a single host species or host genus. Larval stages (metacestodes) from four orders also occur in fish, but are difficult to identify morphologically. Future work should focus primarily on little-studied fish groups and regions with probably undescribed diversity (e.g., the southern USA), combined with integrative taxonomic approaches.

## 1 Introduction

Fish parasitology has a long tradition in North America, and numerous parasitologists have contributed significantly to today’s knowledge of the diversity and biology of freshwater fish parasites (1). The first fish tapeworm in North America was *Bothrimonus sturionis* from sturgeon *Acipenser oxyrinchus* in Ohio (USA), described by Duvernoy in 1842. Most of the knowledge was summarized in monographs (1) or synopses (2, 3). However, this knowledge was based on the morphological identification, which may lead to misidentifications and does not allow a reliable assessment of their phylogenetic relationships and the true range of their host specificity. In addition, research focus on fish parasites slowed down over the last three decades (4). As a result, species lists are outdated, there are numerous taxonomic confusions and a lack of molecular data for most taxa.

Tapeworms (Platyhelminthes: Cestoda) are an interesting and widespread group that provide a suitable model for morphological, physiological, ecological and evolutionary studies due to their unique adaptations to parasitism, their complex life cycles and their close relationship with their hosts. At the beginning of the 20th century, 125 species of adult tapeworms were reported from North American freshwater fishes (1–3, 5).

More recently, fish tapeworms have been intensively studied using methods of integrative taxonomy and phylogenetics, i.e., properly prepared, heat-fixed specimens, including their molecular vouchers of newly collected and museum material. The aim of this review is to provide an updated synthesis of species diversity, phylogenetic relationships, host associations and biogeography, and to highlight important gaps and future research directions in North American freshwater fish tapeworms.

The classification of host specificity follows the classification of Kuchta et al. (6) for helminth parasites of cypriniform fish in North America and Europe, with slight modifications, namely: (i) strict specialist (in only one host species); (ii) congeneric specialist (in several host species of the same genus); (iii) suprageneric specialist (in species of several closely related genera); (iv) low generalist (in hosts of different, not closely related genera); (v) high generalist (in hosts of unrelated fish orders or suborders).

We are well aware of the different categories of host specificity [e.g., (7–9)] and have carefully reviewed the different terms used in the literature. Ultimately, however, we used the categories proposed by Kuchta et al. (6) for tapeworms and ‘monogeneans’ of cypriniform fishes in North America, as they best reflect the taxonomic categories of North American fish hosts of tapeworms, particularly suckers (Catostomidae).

To make the text clearer, authorities and years for parasite and host taxa are omitted throughout the manuscript. The complete authorities of cestode taxa can be found in the Global Cestode Database (10) and those of fish hosts FishBase.^1^

## 2 Species diversity in North America

Since 2000, 21 new species have been described and seven new genera have been proposed (Supplementary Table S1). However, several species recognized by Hoffman (1) have been synonymized (11). In addition, a new subfamily, Essexiellinae, was proposed, which includes the North American tapeworms previously assigned to the Corallobothriinae (12).

**Figure 1:**
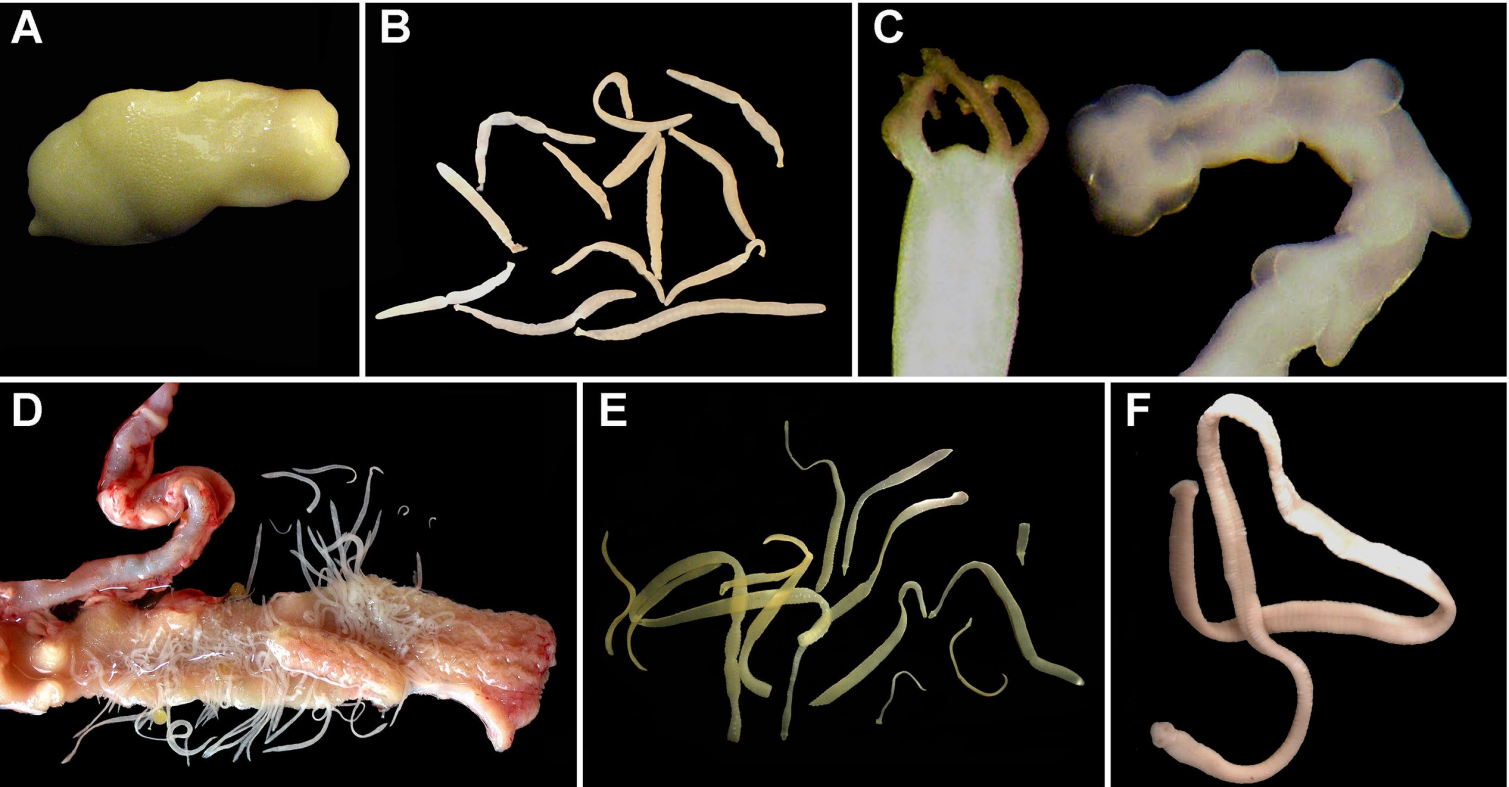
Examples of adult tapeworms (Cestoda) of fhreshwater fishes *in situ*. **(A)** Live *Amphilina foliacea* (Amphilinidea) from the body cavity of *Acipenser ruthenus*, Slovakia (no image of an amphilinidean from North America was available); **(B)** *Cyathocephalus truncatus* (Spathebothriidea) from the intestine of *Salvelinus alpinus*, Norway (no image of a spathebothriidean from North America was available); **(C)** *Haplobothrium globuliforme* (Haplobothriidea) from the intestine of *Amia calva*, Mississippi, USA; **(D)** *Isoglaridacris floriani* in the intestine of *Moxostoma macrolepidotum*, South Carolina, USA; **(E)** *Marsipometra hastata* (Bothriocephalidea) from the intestine of *Polyodon spatula*, Mississippi, USA; **(F)** *Essexiella fimbriata* (Proteocephalidae) from the intestine of *Ictalurus punctatus*, Nebraska, USA.

To date, 130 species of adult tapeworms parasitizing freshwater fish in North America, including Neotropical Mexico, have been described (Figure 1; Supplementary Table S1). Most species belong to the orders Caryophyllidea (72 species) and Onchoproteocephalidea I [here referred to as Proteocephalidae as they are the only family of the former order Proteocephalidea – see (13, 14)] (37 species), followed by Bothriocephalidea (15 species). The individual cestode orders, which are arranged alphabetically, are briefly commented on below.

### 2.1 Bothriocephalidea

**Figure 2:**
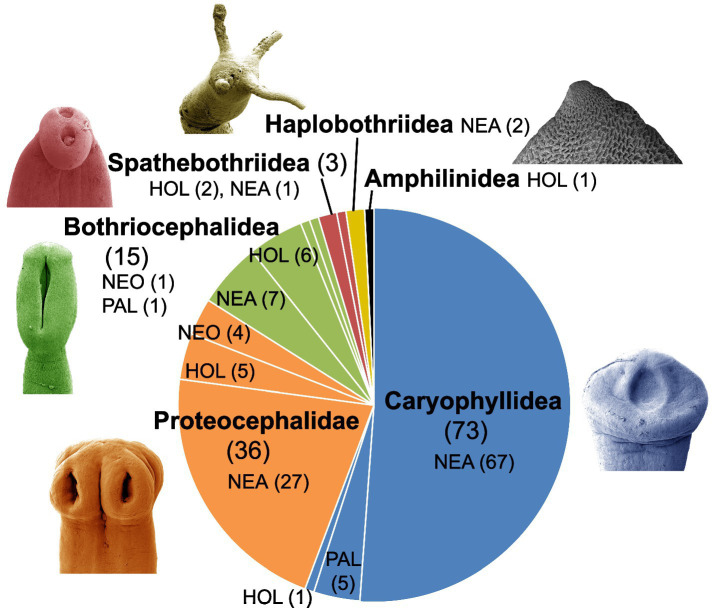
Diversity and zoogeographical distribution of tapeworms (Cestoda) in freshwater fishes of North America.

The tapeworms of the Bothriocephalidea, which are found in North American freshwater fish (in total 15 species - Figure 2), do not form a monophyletic group. Instead, they belong to five separate, not closely related lineages of all three families. Relatively little progress has been made on bothriocephalidean tapeworms in North America since 2000, with the exception of studies on species of *Bothriocephalus*.

The taxonomic history of this species-rich genus is confused due to the generally uniform morphology of its species, the inadequate descriptions of many taxa, and the poor quality of museum specimens (15). One of the most common species, *Bothriocephalus cuspidatus*, has been reported from 33 North American fish species (1, 16). A closer examination of ‘*B. cuspidatus*’ from different hosts based on specimens collected using uniform methods of heat kill and fixation, provided evidence that several separate species have been grouped under the name *B. cuspidatus* in the past (15).

The combination of morphological and molecular data provided evidence for divergence and host specialization patterns in these tapeworms. As a result, *B. kupermani* has been described from sunfishes (*Lepomis* spp.) and three other putative new taxa from *Ambloplites rupestris*, *Micropterus dolomieu* and *M. salmoides* are to be formally described (15, 17). These data, which demonstrate narrow host specificity of individual species, also have implications for faunistic surveys, biodiversity assessments and ecological studies.

A phylogenetic study by Brabec et al. (18) showed that the genus *Bothriocephalus* is an artificial assemblage of several not closely related lineages. Therefore, Scholz et al. (19) proposed the new genus *Bothriocestus* to include freshwater species previously placed in *Bothriocephalus*, including Holarctic *B. claviceps* (type species) and Nearctic *B. cuspidatus* and *B. kupermani*. The establishment of *Bothriocestus* was important to resolve long-standing phylogenetic disagreements, which were further complicated by the fact that the species of the new genus and those of *Bothriocephalus* do not differ by distinct morphological characters (19).

### 2.2 Caryophyllidea

This order includes monozoic tapeworms, i.e., they have a single set of genital organs and no proglottisation (20, 21). Most of the North American species occur in suckers (Catostomidae), where the group has spread relatively recently. These fishes are abundant and widespread in North America. The high level of research activity over the last two decades has enabled the revision of four genera, namely *Archigetes*, *Biacetabulum*, *Glaridacris* and *Promonobothrium* [see (22–27)].

In addition, 17 new species have been described (most of them from the southern USA) in the following genera: *Archigetes* (3 spp.), *Biacetabulum* (5), *Dieffluvium* (1) *Isoglaridacris* (6) and *Promonobothrium* (2). Three new genera have been proposed, namely *Homeomorpha* [recently synonymized with *Isoglaridacris* by (28)], *Megancestus* and *Pseudoglaridacris* (23, 29, 30). These genera represent important phylogenetic lineages and were proposed to reconcile the genus classification with the molecular phylogenetic data.

Currently, 73 species of Caryophyllidea are recognized as valid in North America (Figure 2), which corresponds to an increase of 30% compared to the end of the 20th century. The distribution of all taxa shows that species diversity is increasing from north to south, possibly due to the greater diversity of catostomids in the southern part of the USA. Molecular data suggest that there may be undescribed species. There is a large gap in our knowledge of tapeworms of endemic catostomids in the western part of North America, as shown by the recent description of two new *Isoglaridacris* species from these fishes in northwestern Mexico (28).

### 2.3 Proteocephalidae (Onchoproteocephalidea I)

The taxonomy of this group is probably the most difficult among all freshwater fish tapeworms, especially with regard to the polyphyletic genus *Proteocephalus*. In addition to freshwater fish, species of this group also infect amphibians, reptiles and even a mammal (13). In total, 15 species (instead of 23) of the *Proteocephalus* species-aggregate (= ‘true’ *Proteocephalus* or *Proteocephalus* sensu stricto) were recognized as valid (11). The Proteocephalidae are the second most species-rich group, but the number of recently described species is low (Figure 2; Supplementary Table S1).

The main reasons for this are the poor quality of historical material and the lack of molecular data. In addition, most research activities have focused on the revision of individual genera, and some of newly collected material, which most likely contains new species, is still being analyzed (unpublished data).

Significant achievements were the correction of misidentifications (e.g., a new species from sticklebacks), the clarification of host specificity (a new species of a newly proposed genus specific to gars) and the reorganization of the phylogenetic tree with regard to the genus and subfamily affiliation of tapeworms from ictalurids and gars (11, 12, 31–34). It is likely that future studies on new, properly fixed material, mainly from salmonids, will reveal the existence of further species (35, 36).

In contrast, little progress has been made in clarifying the inadequately clarified taxonomic situation of the *Proteocephalus* species-aggregate of salmonids and minnows. Hoffman (1) reported 11 species from salmonids, but most taxa were synonymised with *P. longicollis* on the basis of morphological studies, but without molecular data (13, 37, 38).

Recently, however, it was found that some of the synonymised species are indeed valid, such as *P. exiguus*, a specific parasite of North American whitefish (*Coregonus* spp.) (35, 39). Scholz et al. (36) commented on the taxonomic status and possible validity of *Proteocephalus* species described from North American whitefish (*Coregonus* spp.), but their taxonomic status cannot be clarified without a detailed morphological and especially molecular assessment of new, properly processed specimens.

In addition to these revisions, new genera for tapeworms specific to ictalurids and bowfin that are endemic to North America were established to reflect phylogenetic findings: (1) *Essexiella* was proposed to include *Corallobothrium fimbriatum* from the channel catfish *Ictalurus punctatus*; and (2) *Laruella* was proposed for *Proteocephalus perplexus* from bowfins, as it is phylogenetically distinct from the ‘true’ *Proteocephalus* species-aggregate (Figure 2) (12, 33).

### 2.4 Three small cestode orders

Of three small orders reported from North American freshwater fishes, namely Amphilinidea (1 species, *Amphilina japonica*, occurs in sturgeon), Haplobothriidea (2 endemic species of *Haplobothrium* occur in bowfin) and Spathebothriidea (3 species occur in a wide range of freshwater and *Diplocotyle olrikii* also in marine fishes), no new species were described (Supplementary Table S1).

Although these orders are represented by only a small number of species in North America, they are important from a phylogenetic perspective (as evolutionary relicts), because of their host specificity (in evolutionarily ancient fish hosts such as sturgeons and bowfin), and because of the overall rarity of most species, with the exception of *Bothrimonus sturionis* and *Cyathocephalus truncatus*, which has been reported from a number of non-closely related fish hosts (1, 3). Moreover, *B. sturionis* was the first fish tapeworm reported in North America (40), but its validity remains unclear (41).

One challenge is the need to review two *Haplobothrium* species that have been reported in the past from the ruddy bowfin (*Amia calva*), following the recent recognition of a second *Amia* species in North America (42). The eyetail (or emerald) bowfin, *Amia ocellicauda*, has a large range and is found in the Great Lakes and Mississippi Basin, while *A. calva* is only distributed in the southeastern United States, particularly in Florida and adjacent states with short rivers leading to the Gulf of Mexico (42). Since *Haplobothrium bistrobilae* occurs exclusively in Florida, it is necessary to genetically compare the *Haplobothrium* tapeworms of both fish species. With strict host specificity (occurrence of *H. globuliforme* in *A. ocellicauda* and *H. bistrobilae* in *A. calva*), the distribution of the tapeworms would help to confirm host speciation.

## 3 Phylogenetic relationships and classification

Over the past two and half decades, great progress has been made in understanding the relationships between tapeworms (43–45), including taxa that parasitise bony fishes (46, 47). These phylogenetic insights are crucial for a better understanding of host–parasite coevolution, biogeography and taxonomy, including a more natural classification of tapeworms. Knowledge of the phylogenetic position and relationships of North American fish tapeworms has improved considerably since 2000. It has been shown that they do not even form a monophyletic group in the same order, with the exception of the native Caryophyllidea (Capingentidae – see below). It is evident that the colonization of North American freshwater fishes by tapeworms has occurred several times independently (47).

Phylogenetic studies have also confirmed the uniqueness of the tapeworm fauna of North American freshwater fishes, as it consists predominantly of species endemic to this continent (Supplementary Table S1). Numerous genera (e.g., *Marsipometra*, almost all native Caryophyllidea, *Corallotaenia*, *Cordicestus*, *Essexiella*, *Laruella* – all Proteocephalidae), subfamilies (e.g., Essexiellinae – Proteocephalidae), families (Capingentidae – Caryophyllidea) and even an order (Haplobothriidea) are endemic to the Nearctic region.

### 3.1 Bothriocephalidea

**Figure 3:**
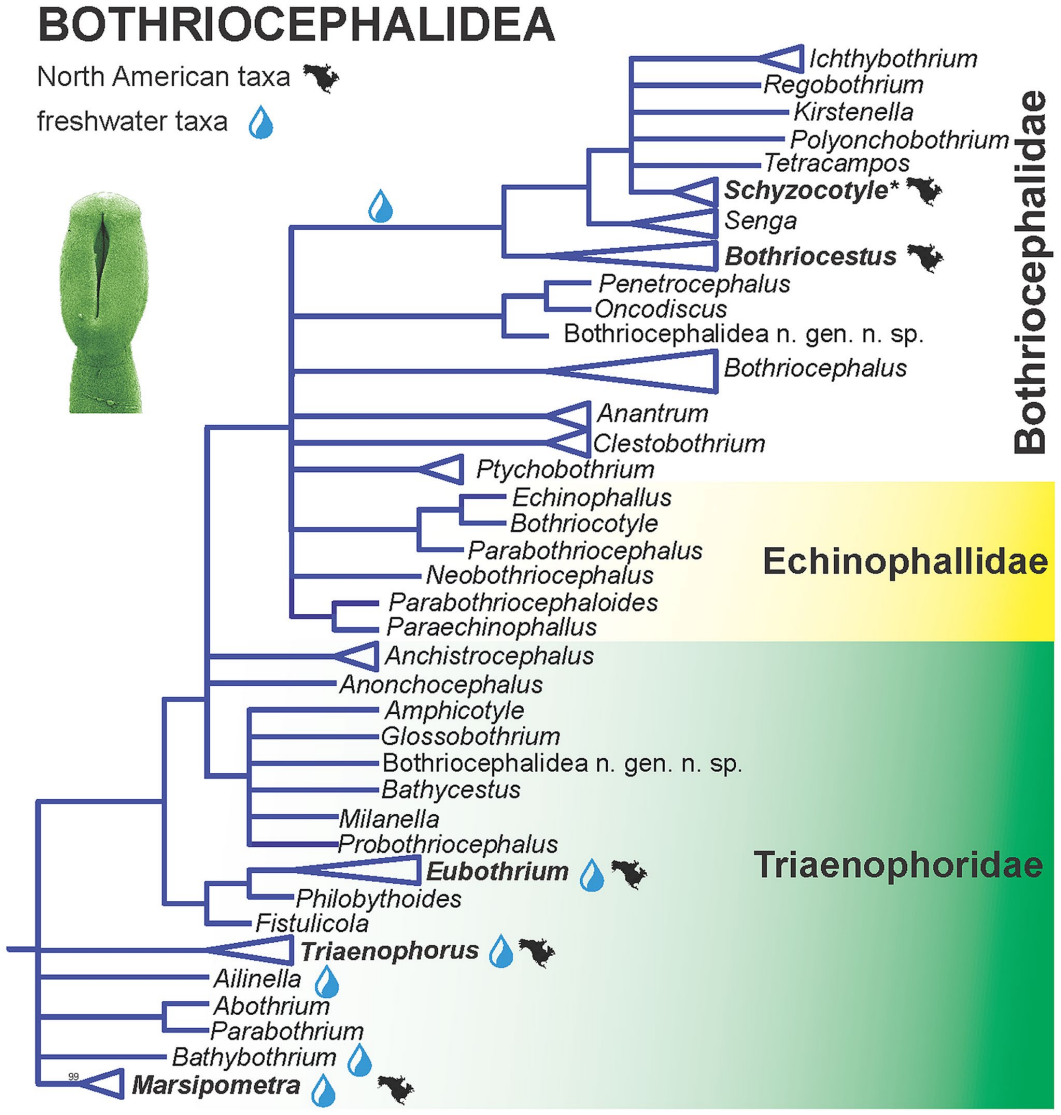
Simplified phylogenetic tree of the Bothriocephalidea inferred from partial 28S rDNA sequences showing the phylogenetic position of North American freshwater fish tapeworms. Branches with support values < 90% were collapsed. Modified from Brabec et al. (18). Asterisk indicates tapeworms non-native (introduced) to North America.

Brabec et al. (18) assessed the relationships of the order, parasitising marine and freshwater bony fishes, using molecular phylogenetic analyses with multiple genes covering about 70% of the currently recognized genera. The new phylogenetic data challenged the morphological classification. In contrast, some patterns in host utilization and environments (freshwater versus marine species) were revealed, but biogeographic patterns are not evident [Figure 1; (18)]. Some of the most basal taxa including North American endemic genus *Marsipometra* from paddlefish *Polyodon spathula* are parasite of freshwater fish, but the origin of the order cannot be resolved based on the available molecular data [Figure 3; (18)].

The order appears to be monophyletic, but consists of non-monophyletic families. The family Bothriocephalidae forms the most derived lineage of the order, with a single freshwater clade I that includes North American taxa, currently classified in *Bothriocestus*, as well as the invasive Asian fish tapeworm, *Schyzocotyle acheilognathi* (syn. *Bothriocephalus acheilognathi*), which is of major veterinary and ecological importance (18, 48). Other North American genera (*Eubothrium* and *Triaenophorus*) belong to the most basal, but non-monophyletic family Triaenophoridae with Holarctic distribution of many freshwater taxa, with the exception of the North American endemic genus *Marsipometra*, *Eubothrium tulipai* from the northern pikeminnow *Ptychocheilus oregonensis* and *Triaenophorus stizostedionis* from the walleye *Sander vitreus*.

### 3.2 Caryophyllidea

Molecular phylogenetic analyses of the Caryophyllidea (and also the Proteocephalidae – see below) do not support traditional taxonomic significance (14, 20). It was based on the position of the reproductive organs (testes, ovary, vitelline follicles and uterus) in relation to the internal longitudinal musculature, which appears to be homoplastic in both groups (13, 21).

As a result, Scholz et al. (49) reorganized the classification of the Caryophyllidea at the family level. Although all existing families remained valid, their composition was significantly changed to reflect the assumed phylogenetic relationships. Most endemic Nearctic taxa are currently placed in the monophyletic family Capingentidae, which represents a relatively recent radiation in North America, including the formation of numerous scolex types and the presence of an external seminal vesicle in most taxa (which is never present in other families). The remaining species belong to a largely Palaearctic group forming the family Caryophyllaeidae. With one exception (*Caryophyllaeides fennica*), all species of this family occurring in North America are invasive and were introduced to America with the common carp (*Cyprinus carpio*), which is not native to North America.

**Figure 4:**
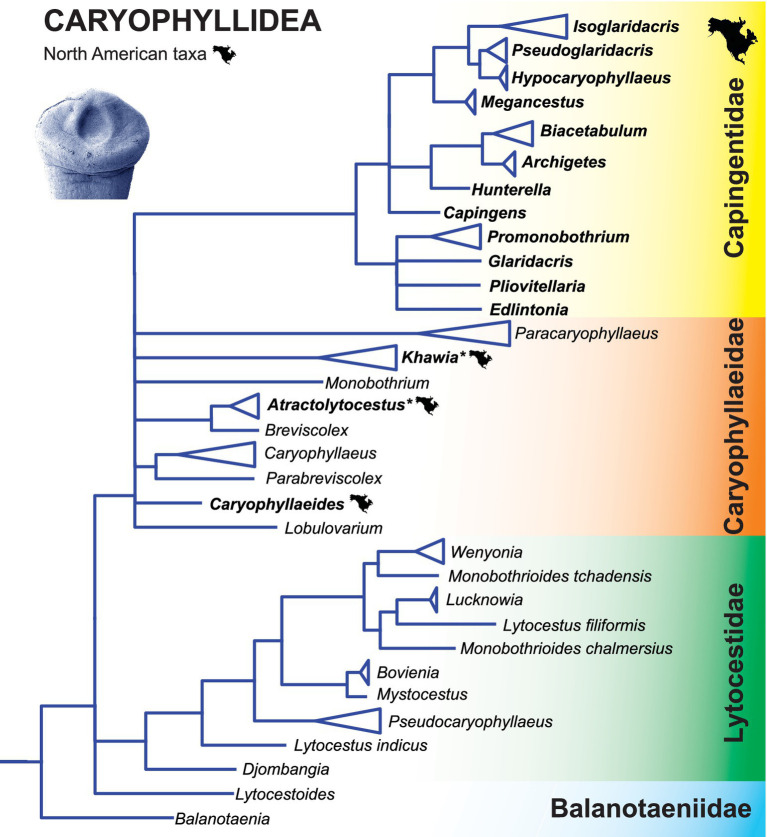
Simplified phylogenetic tree of the Bothriocephalidea inferred from partial 28S rDNA sequences showing the phylogenetic position of North American freshwater fish tapeworms. Branches with support values < 90% were collapsed. Modified from Brabec et al. (18). Asterisk indicates tapeworms non-non-native (introduced) to North America.

The North American taxa (family Capingentidae) are related to those of the Caryophyllaeidae, which occur in the cypriniforms (Cyprinoidei and Cobitoidei) and almost exclusively in the Palaearctic (Figure 4) (49). Interestingly, two species from the native Nearctic leuciscids, *Edlintonia ptychocheila* from the Northern pikeminnow and *Pliovitellaria wisconsinensis* from the golden shiner *Notemigonus crysoleucas*, are the most basal taxa of the North American species of the Capingentidae, which may indicate a host switch in their ancestors from cyprinoids to suckers (Catostomidae) and a subsequent radiation in these hosts (49).

### 3.3 Proteocephalidae (Onchoproteocephalidea I)

Similar to the Caryophyllidea, the previous classification was largely artificial and did not correspond to the actual relationships of the individual groups (13). Molecular phylogenetic studies have not supported the existing subfamilies, most of which are not monophyletic (50, 51). The most species-rich genera, including *Proteocephalus* with species that parasitise freshwater fishes in North America, are polyphyletic, and form several distantly related lineages (13, 51).

The North American fish proteocephalids have been divided into two subfamilies, the Proteocephalinae (species of ‘*Proteocephalus*’), which are characterized by a simple scolex, and the Corallobothriinae, which possess a metascolex, including tapeworms of Nearctic catfishes (Siluriformes: Ictaluridae) (13). The metascolex is the posterior part of a divided scolex consisting of folds of tissue that generally enclose or conceal the suckers [see (10)]. Although the presence of a metascolex has long been used for classification, recent molecular studies show that it is a homoplastic feature that has evolved multiple times independently (13, 50, 51). Therefore, the North American ‘Corallobothriinae’ were transferred to a new subfamily, Essexiellinae, by Scholz et al. (31).

**Figure 5:**
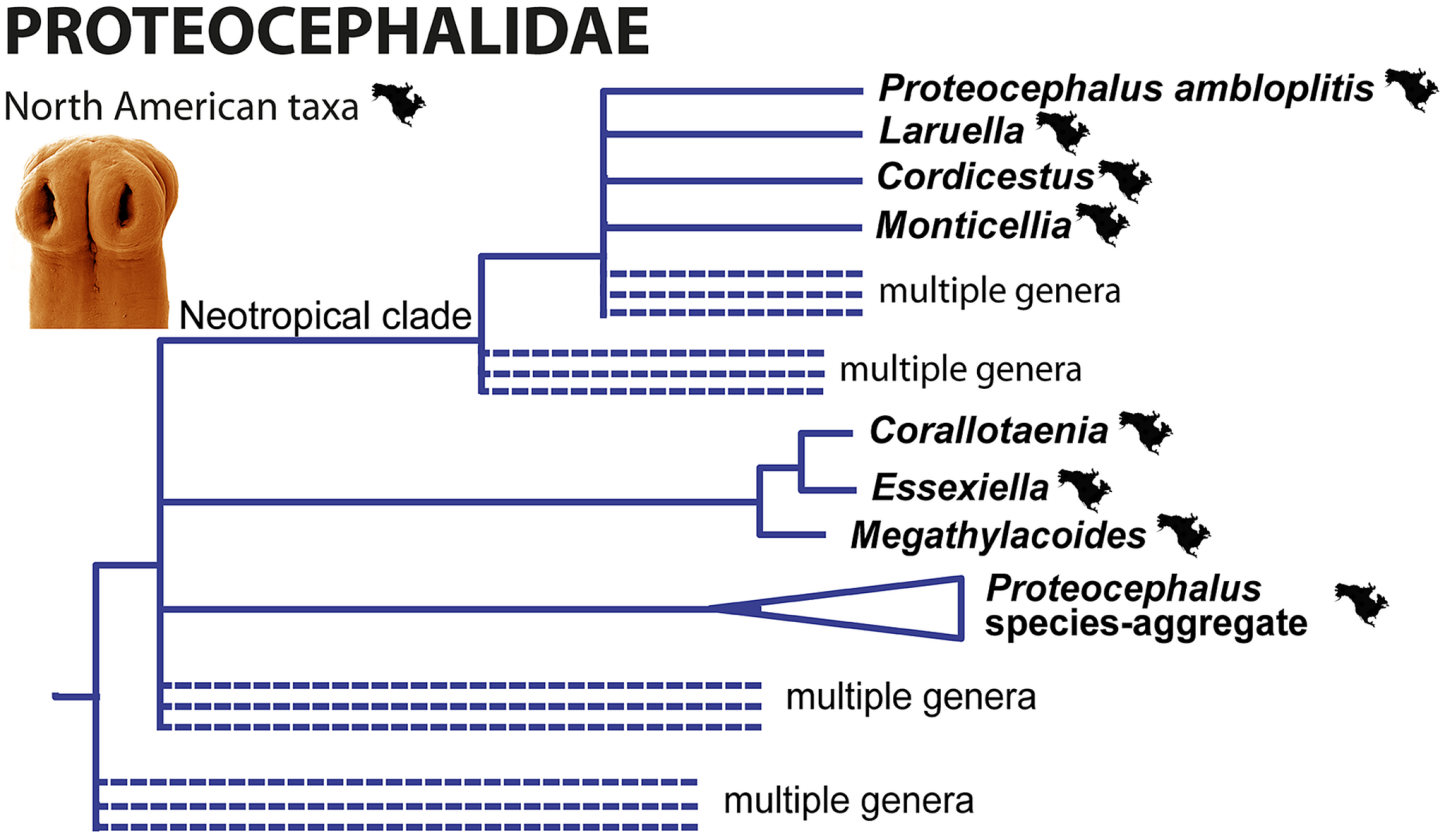
Simplified phylogenetic tree of the Proteocephalidae from partial 28S rDNA sequences showing the phylogenetic position of North American freshwater fish tapeworms. Branches with support values <90% were collapsed.

**Figure 6:**
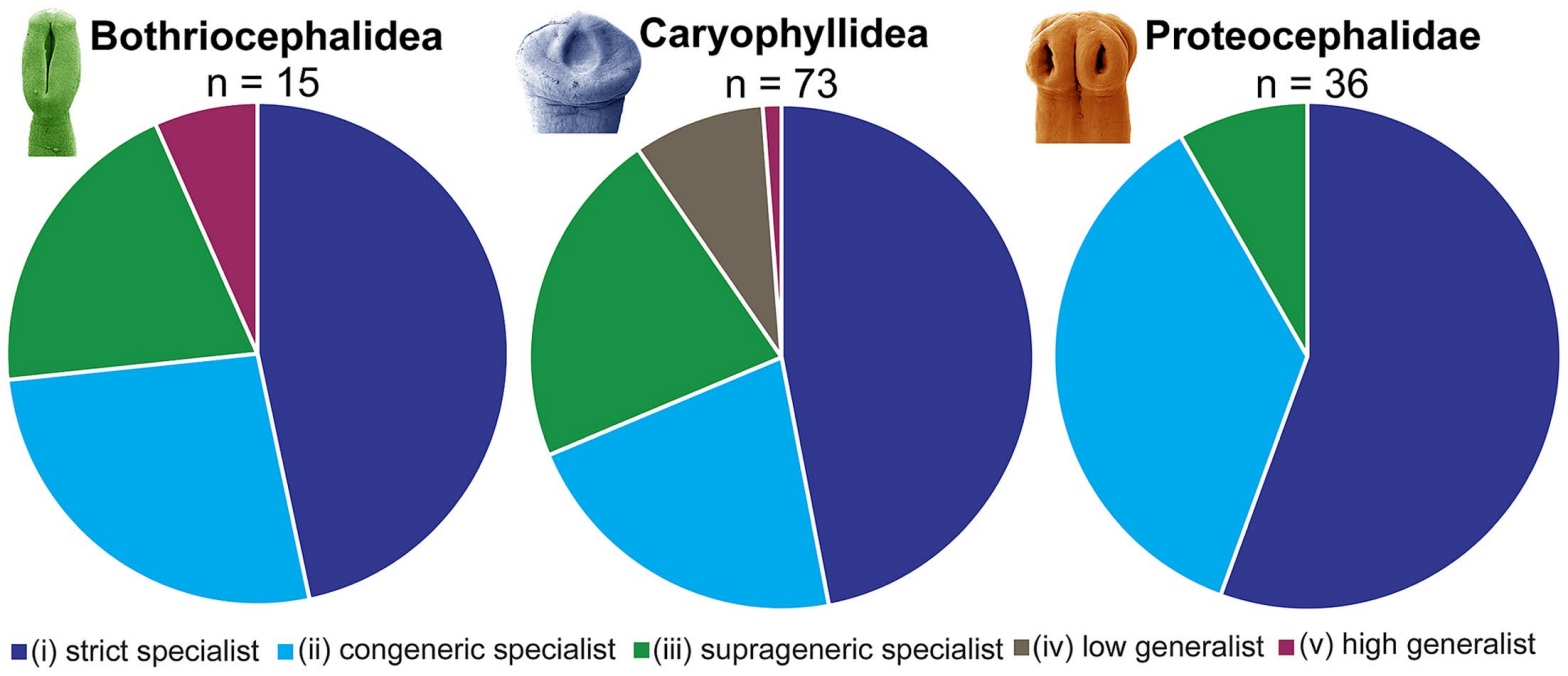
Survey of host specificity of North American freshwater fish tapeworms.

The North American members of the family Proteocephalidae currently belong to at least five different lineages [Figure 5; see also Figures 4–6 in (52)]:

1. *Proteocephalus* species-aggregate (= ‘true’ *Proteocephalus*), corresponding to clade F of de Chambrier et al. (51) (53);
2. Essexiellinae in the ictalurids, i.e., the tapeworms of the genera *Corallotaenia*, *Essexiella* and *Megathylacoides*, corresponding to clade I of de Chambrier et al. (51); the two clades mentioned belong to the relatively basal groups of the Proteocephalidae (51);
3. *Cordicestus* in gars (Lepisosteidae), which belongs to clade D, which is part of the large and unresolved “Neotropical fish” clade D of de Chambrier et al. (51) [see (32)];
4. *Laruella* in the bowfins (Amiidae), which also belongs to clade D of de Chambrier et al. (51);
5. *Proteocephalus ambloplitis* in bass (Centrarchidae), which also belongs to clade D of de Chambrier et al. (51);
6. *Monticellia ophisterni* in obscure swamp eel (Synbranchiformes) from Neotropical Mexico, which also belongs to clade D (Figure 5).

The phylogenetic position of *Testudotaenia testudo*, a parasite of turtles and possibly accidentally found in bowfin, is not clear (54).

Interestingly, the oldest, relict fishes such as gars (Lepisosteiformes) and bowfin (Amiiformes), are definitive hosts of relatively recently evolved tapeworms, while the most recently evolved teleosts such as centrarchids and percids harbor tapeworms belonging to relatively basal Proteocephalidae (32, 51). This indicates several host switches of the Nearctic proteocephalids and their ancestors.

### 3.4 Small cestode orders

Recent molecular phylogenetic studies confirmed the monophyly and validity of all three small cestode orders (Amphilinidea, Haplobothriidea and Spathebothriidea) found in North American freshwater fishes, as circumscribed by Khalil et al. (55). Phylogenetic studies confirmed the basal position of the monozoic, non-strobilised Amphilinidea, which is one of the two earliest diverging orders of tapeworms (45). Overall, these three small orders – possibly due to their low veterinary importance and small number of species – remain poorly studied and require future investigation. No further changes have been made to the classification, with the exception of new molecular data and revision of the Spathebothriidea (41).

## 4 Parasite–host associations (host specificity)

Adequate knowledge of the host range of parasites, i.e., host specificity, is crucial for ecological, epidemiological and evolutionary implications. Based on published data, particularly those compiled by Hoffman (1), a relatively broad host specificity has been assumed for many freshwater fish tapeworms in North America. However, a critical review of the host specificity of tapeworms of North American freshwater fish allowed their categorization into one of five groups according to the range of host specificity: (i) 59 species, i.e., 45%, are strict specialists (they live in only one host species); (ii) 36 species (28%) are congeneric specialists (they live in several host species of the same genus); (iii) 24 species (18%) are suprageneric specialists (they live in species of several closely related genera); (iv) 7 species (6%) are low generalist (they live in hosts of different, not closely related genera); and (v) 4 species (3%) high generalist (they live in hosts of unrelated fish orders or suborders) (Supplementary Table S1).

It is evident that the vast majority of North American freshwater fish tapeworms exhibit narrow host specificity, with almost three quarters specializing in a single host species or host genus. In contrast, none of the high generalists are endemic to North America. These high generalists are the invasive (probably Palaearctic) *Schyzocotyle acheilognathi* (Bothriocephalidea), the non-native (Palaearctic) *Archigetes sieboldi* (Caryophyllidea), and the Holarctic *Cyathocephalus truncatus* and *Diplocotyle olrikii* (both Spathebothriidea) (Supplementary Table S1).

Interestingly, species of all six orders are generally found in different groups of North American fish, with the Caryophyllidea in particular being the only tapeworms found in suckers (Catostomidae) (rare records of the invasive *Schyzocotyle acheilognathi* are not considered). It is relatively rare for adult tapeworms of different orders to be found in the same fish, such as the pike, which harbors *Proteocephalus pinguis* (Proteocephalidae), *Triaenophorus* spp. (Bothriocephalidea) and *Cyathocephalus truncatus* (Spathebothriidea), bowfin, which is the definitive host of *Haplobothrium* spp. (Haplobothriidea) and *Laruella perplexa* (Proteocephalidae), salmonids (hosts of species of three orders), and northern pikeminnow, which harbors *Eubothrium tulipai* (Bothriocephalidea) and *P. torulosus* (Proteocephalidae) (Supplementary Table S1). Current knowledge of host specificity also reflects the intensity of sampling, as some popular sport fish such as centrarchids (smallmouth and largemouth bass) and walleye have been more intensively sampled for parasites compared to other fish of lesser commercial interest (4).

The latest findings on the host specificity of the individual cestode orders are explained below (Figure 6; see also Supplementary Table S1).

### 4.1 Bothriocephalidea

This order includes 15 species with a variable range of host specificity: 7 strict specialists (e.g., the bothriocephalideans *Eubothrium tulipai* and *Triaenophorus stizostedionis*), 4 congeneric specialists, 3 suprageneric specialist and 1 high generalist (invasive, non-native *Schyzocotyle acheilognathi*) (Figure 6; see also Supplementary Table S1).

*Bothriocestus cuspidatus* has been reported from 33 species of freshwater fishes from 13 families in 12 orders (1, 16). However, Choudhury and Scholz (15) showed that *B. cuspidatus* does not exhibit such broad host specificity. Instead, several species specialized on their own definitive hosts were grouped together as *B. cuspidatus*; they are congeneric or suprageneric specialists (15, 18). Several hosts have been reported based on immature or juvenile tapeworms that cannot be reliably identified by morphology alone.

*Schyzocotyle acheilognathi*, commonly known as Asian fish tapeworm, is a highly invasive cestode that infects a wide range of freshwater fish (> 300 species), especially cyprinoids (minnows in North America) (56). The parasite poses a significant threat to aquaculture and the biodiversity of native fish worldwide, including North America (57). It is most pathogenic in newly acquired host species, which appears to be the case in Australia, Europe and North America (1, 58, 59). The species was transferred to genus *Schyzocotyle*, based on a molecular phylogenetic study by Brabec et al. (18). The two species of this resurrected genus are characterized by a heart-shaped scolex with narrow, deep bothria (57).

The North American *Eubothrium* species have very different host patterns. The two species most commonly parasitising salmonids, *Eubothrium crassum* and *E. salvelini*, were reported by Hoffman (1) from at least 16 fish species from different families and orders. However, *E. crassum* mainly infects trout and salmon (*Salmo* spp.), while *E. salvelini* is typically associated with charr (*Salvelinus* spp.) and whitefish (*Coregonus* spp.). However, both species have been detected in rainbow trout (*Oncorhynchus mykiss*) in Europe (60), and this may also be the case in other Pacific salmon of the genus *Oncorhynchus*, although this remains to be confirmed. In contrast, the two remaining *Eubothrium* species in North American freshwater fishes, *E. rugosum* from the burbot, *Lota lota*, and *E. tulipai*, from the northern pikeminnow, are strict specialists [(61); Supplementary Table S1].

There are numerous records of bothriocephalideans in atypical or unusual fish hosts [see (1)], but these should be examined critically as they are likely accidental infections or postcyclic parasitism.

### 4.2 Caryophyllidea

The Caryophyllidea comprise 73 species, the vast majority of which exhibit narrow host specificity: 29 strict specialists (e.g., six *Isoglaridacris* species, five *Biacetabulum* species and all three *Penarchigetes* species), 18 congeneric specialists, 18 suprageneric specialists, 7 low generalists and 1 high generalist (*Archigetes sieboldi* in the Cypriniformes and Atheriniformes) (Supplementary Table S1). The first two categories with the narrowest host specificity (tapeworms living in a single host species or genus) account for almost two thirds (64%) of all Caryophyllidea in North American freshwater fish (Figure 6).

In contrast, some species of *Promonobothrium* and *Pseudoglaridacris* have a broader range of definitive hosts and are found in fish of different genera or tribes, but rarely in fish of different subfamilies (Catostominae versus Ictiobinae) [(6, 22, 23); Supplementary Table S1].

### 4.3 Proteocephalidae (Onchoproteocephalidea I)

Their host specificity is not sufficiently known, mainly because of the persisting taxonomic problems in this group and the existence of numerous unconfirmed reports of atypical or dubious hosts. The present analysis shows that of the 36 species of Proteocephalidae, 20 are strict specialists, 13 are congeneric specialists and 3 are suprageneric specialists. No species was categorized as low or high generalist (see Supplementary Table S1). Regarding the proportion of species in the category with the highest host specificity (strict specialist), more than half (56%) of the Proteocephalidae belong to this category (compared to 50% in the Bothriocephalidea, albeit with a low total number of species, and 40% in the Caryophyllidea) (Figure 6).

Although recent studies have provided new data on the actual host specificity of many species, there are still large gaps (36). An example of this is the so-called bass tapeworm, *Proteocephalus ambloplitis*, adults of which have been reported from a number of phylogenetically distant fish groups, notably bass, bowfin and gars. However, it appears that at least two species, including *P. australis* from gars, have been confused with *P. ambloplitis* (32). The same is true for metacestodes with a large apical glandular organ, which have been consistently (and automatically) identified as *P. ambloplitis* and are found in a wide range of numerous fish groups (1, 3). However, they actually belong to two or more different species (32, 62).

Nevertheless, most species of the Proteocephalidae appear to have a narrow host specificity as tapeworms of other orders, including several strict specialists such as species of the *Proteocephalus* species-aggregate of sticklebacks (Gasterosteidae), American yellow perch (*Perca flavescens*) and cavefish (Amblyopsidae) (11, 34, 53).

*Laruella perplexa* is a specific parasite of bowfin and has been morphologically and genetically characterized, albeit with a relatively complex genetic structure of isolates from different parts of North America (33). Therefore, the presence of *L. perplexa* (or another closely related and morphologically similar species) in the two recently distinguished bowfin species [see (42)] should be confirmed.

A recent study of tapeworms of gars (Lepisosteidae), ancient and relict group of fish, confirms a narrow host specificity and restricted distribution of these proteocephalids, with closely related species occurring in different zoogeographical regions, i.e., Nearctic and Neotropical (32).

### 4.4 Small cestode orders

*Amphilina japonica* (syn. *A. bipunctata*) (Amphilinidea), the only Amphilinidea from North America, is a specialist and occurs in the body cavity of the white sturgeon, *Acipenser transmontanus* in North America and of *Sinosturio mikadoi* in Japan. However, molecular data are still lacking to confirm the validity of this species, as it closely resembles *A. foliacea* from Eurasian sturgeons (63).

*Haplobothrium globuliforme* (Haplobothriidea) is a typical parasite of bowfin but has also been reported from the American eel, *Anguilla rostrata*. The latter fish was most likely only an accidental or postcyclic host. Similarly, a very rare *H. bistrobilae*, the validity of which remains to be confirmed, has been found in *A. calva* and in brown bullhead, *Ameiurus nebulosus*, although the latter host is questionable.

Two species of the relict order Spathebothriidea [see (41)] have a relatively broad spectrum of definitive hosts. *Bothrimonus sturionis*, a parasite of sturgeons (Acipenseridae), has been reported from 13 other fish hosts, including species from three salmonid genera, in Canada (1, 3). However, these reports are most likely a confusion with the morphologically similar *Diplocotyle olrikii* from marine and anadromous fish, as members of the genus *Bothrimonus* appear to be specific parasites of sturgeons (41, 64). The Holarctic *Cyathocephalus truncatus* was found in 16 species from six orders. With the exception of *B. sturionis*, the North American freshwater species of the order Spathebothriidea are high generalists.

## 5 Zoogeography

### 5.1 Endemism of North American fish tapeworms

The tapeworm fauna of the freshwater fishes of North America is unique and peculiar, with a high degree of endemism (4/5 of the species – Figure 2). Nearctic elements dominate (96 species, i.e., 79%), with a small proportion of Holarctic taxa (15, i.e., 12%) and a negligible number of Neotropical taxa (5 in Mexico, i.e., 4%) and species introduced to North America with their non-native fish hosts, in particular the common carp (6, i.e., 5% – Figure 3), which harbors five tapeworm species in North America (1 of the Bothriocephalidea, 4 of the Caryophyllidea).

The only species of Caryophyllidea with a circumboreal distribution, *Caryophyllaeides fennica*, was recently found by Oros et al. (65) in the chiselmouth *Acrocheilus alutaceus*, an endemic cyprinid in the northwestern Nearctic (Oregon). A very low genetic divergence between *C. fennica* representatives from the Palaearctic and the Nearctic indicates a relatively recent colonization of the Nearctic by this cestode across the Beringian land bridge (65).

There are at least 14 species (only 11% of all species) with a Holarctic distribution, namely *Amphilina japonica* (Amphilinidea), *Bothriocestus claviceps*, *Eubothrium crassum*, *E. rugosum*, *E. salvelini*, *Triaenophorus crassus*, *T. nodulosus* (Bothriocephalidea), *Proteocephalus ambiguus*, *P. filicollis*, *P. macrocephalus*, *P. tetrastomus*, *P. torulosus* (Proteocephalidae), *Cyathocephalus truncatus* and *Diplocotyle olrikii* (Spathebothriidea) (11, 19, 66, 67).

### 5.2 Distribution in North America

The vast majority of species occur in the USA (121 species), while less than half of the species diversity was found in Canada (58 species) and Mexico (14 species), including nine species in the Nearctic part of the country and only five Neotropical taxa (Supplementary Table S1).

As far as the main hydrological regions of North America are concerned, it is not possible to provide detailed and reliable data, as there are numerous misidentifications of individual records. It can roughly be summarized that the Bothriocephalidea and the Proteocephalidae have a similar species diversity in Canada and the USA, with the number of species increasing slightly from Canada toward the south of the USA, while very few species of these orders have been reported from Mexico. In the Caryophyllidea, there is a clear increase in species diversity from Canada (20 species) toward the south and south-east of the USA with the highest number of species. However, the freshwater fish tapeworms in Canada, the southern USA and Mexico are still little studied despite the high diversity of fish.

## 6 Metacestodes (tapeworm larvae)

Metacestodes can also be found common in freshwater fish, and some species can be harmful to their fish hosts, particularly the plerocercoids that migrate through their tissues and internal organs (59, 68). For example, plerocercoids of *Dibothriocephalus* spp. encapsulate in the viscera or body cavity and can cause compression and displacement of pancreatic and testicular tissue in heavily infected hosts. They can also cause liver necrosis, necrosis of the myofibrils near the parasites and hypertrophy of the connective tissue. Mortality of small fish due to hemorrhage may be caused by migrating plerocercoids (59, 68–71).

Tapeworm larvae of the following five orders parasitise in North American freshwater fish: Bothriocephalidea, Diphyllobothriidea, Haplobothriidea, Proteocephalidae and Cyclophyllidea (1, 47). The most important are plerocercoids of the genera Dibothriocephalus (formerly in Diphyllobothrium), Triaenophorus and Proteocephalus ambloplitis (sensu stricto), which can invade the body cavity or internal organs including the musculature of fish and encapsulate in the viscera or muscles (1, 49, 69, 70).

Cestode larvae can be localized in different organs, i.e., in the abdominal cavity (larvae of *Dibothriocephalus*, *Ligula* and *Schistocephalus*; all Diphyllobothriidea), in the liver and muscles (e.g., larvae of *Triaenophorus*; Bothriocephalidea), in the gall bladder (*Valipora*) and in the liver (*Paradilepis*; both Cyclophyllidea) (1, 68). Metacestodes can also be very numerous and their correct species identification is important for epizootiological, ecological and faunistic studies.

Metacestodes generally do not have genital organs (or only their primordia), which are crucial for species identification. In addition, their scolex may differ from that of the adult worms, making their reliable identification difficult or impossible, as does the simultaneous occurrence of larvae of different species in the same hosts or even in the same organs of these hosts (e.g., *Dibothriocephalus dendriticus* and *D. ditremus*). As a result, many tapeworms have been misidentified, such as the metacestodes of *Protepocephalus australis* from gars as *P. ambloplitis* (32). However, correct species identification is important from an epizootiological or epidemiological point of view mainly for zoonotic parasites such as some Diphyllobothriidea (72).

In addition, the records of adult tapeworms and metacestodes in the older literature, including the monograph by Hoffman (1), cannot always be distinguished, as they are reported exclusively as parasite–host records with no indication of developmental stage or maturity. Therefore, the information in the literature should be treated with caution, especially with regard to the actual occurrence of some larvae in numerous fish hosts.

The application of molecular approaches (DNA barcoding or the use of microsatellites in the case of morphologically indistinguishable metacestodes occurring in one and the same fish – (73) seems to have solved most of the above-mentioned problems in the reliable identification of metacestodes (73). However, this approach cannot be applied when the number of metacestodes is high, as it is costly, time-consuming and requires appropriate facilities.

Most conspicuous are plerocercoids of the genera *Ligula* and *Schistocephalus* (Diphyllobothriidea), which grow to large size in the body cavity of cypriniforms and sticklebacks and can adversely affect the growth, sexual maturation and motility of heavily infected fish (58, 71). Plerocercoids of some species of the genus *Dibothriocephalus* (mainly *D. nihonkaiensis* in Pacific salmon and *D. latus* in pike, perch and burbot) are a source of infection for humans and cause diphyllobothriosis (72).

## 7 Conclusion and avenues for future research

Since 2000, important taxonomic updates have been made, particularly in the Caryophyllidea, Proteocephalidae and Bothriocephalidea, and some knowledge gaps have been filled, particularly through a more detailed assessment of host specificity and distribution. The importance of molecular insights in studies is beyond question, as evidenced by broad molecular phylogenies of the major groups of fish tapeworms (43, 47). New data on species diversity, host–parasite relationships and distribution will contribute to more reliable ecosystem monitoring, but also to fisheries management (identification of potentially pathogenic taxa, including metacestodes) or zoonotic risk, which is mainly limited to broad fish tapeworms (Diphyllobothriidea) (72).

The key factor that has enabled this progress in our understanding of the species composition and phylogenetic relationships is integrative taxonomy, i.e., the combination of morphological, molecular and ecological data, including voucher specimens of molecular samples, i.e., holo- and paragenophores [see (74)]. However, a prerequisite for obtaining a broad range of information required for taxonomic, evolutionary and biogeographical conclusions is the presence of properly fixed and adequately processed specimens (75). Much valuable material has been lost through the use of inappropriate methods, such as parasitological examination of dead hosts leading to rapid decomposition of tapeworms, fixation under pressure causing unnatural deformation and expansion, or the use of cold (unheated) fixatives leading to unnatural contraction and deformation of specimens.

For future ecological and zoogeographical analyses, it is essential to critically examine the actual host specificity of individual taxa, preferably by genotyping and depositing specimens from atypical or unusual hosts. The use of molecular markers (DNA genotyping) is unavoidable, especially for metacestodes. Regarding suitable molecular markers, it is recommended to use both nuclear (28S rDNA or LSU) and mitochondrial (*cox*1) gene sequences for genotyping. Genomic data could be used for the development of future tools for diagnosis and epidemiological surveys, including environmental DNA screening (eDNA) for presence of life cycle stages of broad tapeworms in the environment (72).

The top priority for future research is undoubtedly the integration of different methodological approaches and the application of appropriate methods of sample processing and analysis. Below are some more specific points that are important for further understanding the biodiversity, relationships and distribution of tapeworms of North American freshwater fishes, but also for ecosystem monitoring, fisheries management and zoonotic risk assessment.

1. International cooperation, including the participation of experts in individual groups of fish tapeworms in field surveys, processing of samples and their analysis in the laboratory.
2. Targeted sampling of insufficiently studied hosts, in insufficiently studied regions and in areas where new species are suspected, e.g., the southern Mississippi basin and the Pascagoula River.
3. Only fresh fish hosts should be examined; parasite samples from frozen hosts are only suitable for DNA genotyping, but not for their morphological evaluation.
4. The use of appropriate methods for sample processing, especially heat fixation of fresh parasite material (75).
5. Vouchering specimens (especially those used for molecular studies) and their deposition in recognized parasitological collections and museums (74).
6. The application of integrative taxonomy methods, including genotyping of specimens.
7. Critical review of records of parasites from unusual or atypical fish hosts to obtain a more reliable assessment of actual host specificity; this requires the deposition of voucher specimens in parasitological collections for verification.
8. The transfer of taxonomic knowledge to a new generation of parasitologists, as expertise in parasite identification is currently being lost (courses or workshops to promote integrative taxonomy as a background for other studies organised).
9. Genotyping of fish hosts from taxonomically difficult groups (e.g., salmonids or cypriniforms).

With regard to the individual orders of fish tapeworms, the tasks considered the most difficult by the present authors are listed in order to promote future systematic and ecological research, with the greatest gaps still existing in the Proteocephalidae and Caryophyllidea:

Bothriocephalidea: Current species diversity of *Bothriocestus* and *Bothriocephalus*, and host specificity of *Eubothrium* tapeworms from salmonids, including reliable and easy identification of their plerocercoids [see e.g., (73)].

Caryophyllidea: Revision of the tapeworms parasitising *Catostomus* suckers in western North America, especially those of *Glaridacris*, and a critical assessment of the diversity and host specificity of the tapeworms of redhorse (*Moxostoma* spp.), buffalo fish (*Ictiobus*) and chubsucker (*Erimyzon*).

Proteocephalidae: Better understanding of species diversity in salmonids, revision of the subfamily Essexiellinae of ictalurids, in particular circumscription of *Corallotaenia* of bullheads (*Ameiurus*), phylogenetic relationships of species from cavefish and clarification of species diversity of *Laruella* in the recently split species of bowfins.

Small cestode orders: Species composition of *Haplobothrium*, with emphasis on its occurrence in the recently resurrected *Amia ocellicauda*. The validity of *Amphilina japonica* and *Bothrimonus sturionis* from sturgeons should also be confirmed by molecular methods.

A major challenge for the near future is also to compile the available data into comprehensive accounts, including monographs and taxonomic revisions with keys to species identification, which will serve as a solid basis for ecological and evolutionary studies. The life cycles of most fish tapeworms in North America are still poorly understood, and data on their impact on fish hosts are inadequate. Despite these gaps and limited knowledge, North American freshwater fish tapeworms undoubtedly provide valuable models for the study of host specificity, coevolution and phylogenetic relationships. Some of them, including invasive species such as the Asian fish tapeworm, are also important for fisheries and conservation biology, and broad fish tapeworms include potentially zoonotic taxa. Future efforts should focus on integrative taxonomic approaches, international collaboration and applied research linking parasite diversity to ecosystem and fish health.

## Data Availability

The original contributions presented in the study are included in the article/Supplementary material, further inquiries can be directed to the corresponding author.

## Footnotes

^1^https://www.fishbase.se/search.php; (Accessed on July 1, 2025).
